# Medical Association of Georgia

**Published:** 1885-05-20

**Authors:** 


### MEDICAL ASSOCIATION OF GEORGIA.

The Association met in Savannah, Georgia, on the 15th of April.

Addresses of welcome were delivered by Dr. W. H. Elliott and Major Lester, of Savannah. Dr. Holmes, of Rome, responded on behalf of the Association.

The meeting was not so largely attended as usual. There were about fifty members present at the opening.

Dr. A. W. Calhoun introduced President Eugene Foster, who delivered the Annual Address.

The Committee on Expert Testimony and Anatomical Material failed to report, but the Secretary stated, at their request, that the bills were in the hands of a committee of theLegislature, which had reported favorably upon them.

The Committee on Publication reported that they had found difficulty in publishing the Transactions for the want of funds. Three hundred and fifty copies had been published at a cost of five hundred and ninety-nine dollars and sixty-four cents. Only two hundred and fifty-three dollars in dues had been collected, which amount had been supplemented, by advertisements and sales of Transactions, to a sum amounting to four hundred and seventy-three dollars. A committee was appointed to consider the question of having the Transactions published in *journal form*, or, rather, to consider a proposition by Dr. Gray, the Secretary, and the Editor of the Atlanta Medical Journal, to publish the Transactions in that journal. The committee was composed of the following names: Drs. T. J. Charlton, W. B. Parks, S. B. Hawkins, W. B. Cheatham, L. B. Alexander, T. M. Holmes, J. J. Hill, L. G. Hardman, E. C. Goodrich. They reported as follows:

"The committee recommend that the Association accept the proposition of the Atlanta Medical and Surgical Journal, viz.: The Atlanta Journal proposes to publish the Transactions in the Journal for one year, furnish each member with the Journal and pay all expenses of the Secretary and Treasurer's offices (except their expenses to and from the meetings) for $2.00 per member. We also recommend that the members shall not receive the Journal until their dues are paid in full."

The following are the officers and members of committees for the ensuing year:

President—R. J. Nunn, Savannah.

First Vice-President—L. B. Alexander, Forsyth.

Second Vice-President, T. F. Walker, Cochran.

Censor—E. H. Richardson, Cedartown.

Committee on Publication—James A. Gray, E. C. Goodrich, Robt. Battey, H. V. M. Miller, W. D. Bizzell, J. P. Logan.

Committee on Necrology—K. P. Moore, A. G. Whitehead, G. W. Holmes, J. S. Todd.

Committee on Prize Essays—R. J. Nunn, Robt. Battey, W. A. Love, H. F. Campbell, Eugene Foster.

Committee of Arrangements—E. C. Goodrich, J. M. Hull, C. W. Hickman, Eugene Foster, W. H. Doughty, jr., T. R. Wright.

### SECTIONS—FIRST DISTRICT.

Practice—W. Duncan, K. A. Quarterman, J. J. Waring.

Surgery—W. H. Elliott, R. G. Norton, G. H. Stone.

Gynecology—R. B. Harris, E. W. Lane, J. Weichselbaum.

SECOND DISTRICT.

Practice—T. A. Chappell, W. B. Cheatham, L. B. Bouchelle.
Surgery—T. M. McIntosh, W. S. Wood, J. G. Hopkins.
Gynecology—A. E. Taylor, B. R. Doster, P. S. Hilsman.

THIRD DISTRICT.

Practice—A. A. Smith, Chas. Hicks, E. K. Bozeman.
Surgery—S. B. Hawkins, J. D. Herman, A. K. Taylor.
Gynecology—T. F. Walker, J. F. Fielder, R. O. Ingram.

FOURTH DISTRICT.

Practice—C. D. Smith, W. A. Pool, W. W. Fitts.
Surgery—J. T. Slaughter, J. A. Beasly, J. F. Cole.
Gynecology—A. W. Griggs, Geo. B. Grimes, Paul Faver.

FIFTH DISTRICT.

Practice—W. S. Elkin, W. H. Pool, Henry Gaither.
Surgery—James A. Gray, W. B. Parks, R. H. Taylor.
Gynecology—W. A. Love, J. McF. Gaston, T. S. Powell.

SIXTH DISTRICT.

Practice—C. H. Hall, J. C. Solomon, W. O'Daniel.
Surgery—Lewis Harris, H. McHatton, A. J. Williams.
Gynecology—K. P. Moore, T. O. Powell, L. B. Alexander.

SEVENTH DISTRICT.

Practice—T. M. Holmes, W. S. Kendrick, F. R. Calhoun.
Surgery—W. B. Wells, J. R. S. Holmes, E. H. Richardson.
Gynecology—Robert Battey, J. W. Clements, C. P. Gordon.

EIGHTH DISTRICT.

Practice—J. J. Hill, A. A. Bell, J. C. Pope.
Surgery—S. C. Benedict, E. D. Alfriend, R. B. Nisbet.
Gynecology—John Gerdine, A. Moody Burt, G. W. Mulligan.

NINTH DISTRICT.

Practice—J. B. Pendergrass, J. H. Goss.
Surgery—L. G. Hardman, W. J. Bush, R. L. Harris.
Gynecology—J. W. Bailey, W. A. Watson.

TENTH DISTRICT.

Practice—S. D. Brantley, A. H. Baker, J. M. Kelly.
Surgery—DeSaussure Ford, T. R. Wright, M. G. Hatch.
Gynecology—H. F. Campbell, W. H. Doughty, A. G. Whitehead.

The next meeting of the Association will be held on the 3d Wednesday in April, 1886, at Augusta, Ga.

